# Blood biomarkers of neurodegeneration associate differently with amyloid deposition, medial temporal atrophy, and cerebrovascular changes in *APOE* ε4-enriched cognitively unimpaired elderly

**DOI:** 10.1186/s13195-024-01477-w

**Published:** 2024-05-18

**Authors:** Mikko Koivumäki, Laura Ekblad, Juan Lantero-Rodriguez, Nicholas J. Ashton, Thomas K. Karikari, Semi Helin, Riitta Parkkola, Jyrki Lötjönen, Henrik Zetterberg, Kaj Blennow, Juha O. Rinne, Anniina Snellman

**Affiliations:** 1 Turku PET Centre, Turku University Hospital, University of Turku, Turku, Finland; 2 Department of Geriatric Medicine, Turku University Hospital and University of Turku, Turku, Finland; 3 Department of Psychiatry and Neurochemistry, Institute of Neuroscience & Physiology, Sahlgrenska Academy, University of Gothenburg, Mölndal, Sweden; 4 Centre for Age-Related Medicine, Stavanger University Hospital, Stavanger, Norway; 5 Department of Old Age Psychiatry, Maurice Wohl Clinical Neuroscience Institute, King’s College London, London, UK; 6 NIHR Biomedical Research Centre for Mental Health & Biomedical Research Unit for Dementia at South London & Maudsley NHS Foundation, London, UK; 7 Department of Psychiatry, University of Pittsburgh, Pittsburgh, PA USA; 8 Department of Radiology, Turku University Hospital, University of Turku, Turku, Finland; 9 Combinostics Ltd, Tampere, Finland; 10 Wallenberg Centre for Molecular and Translational Medicine, University of Gothenburg, Gothenburg, Sweden; 11 Clinical Neurochemistry Laboratory, Sahlgrenska University Hospital, Mölndal, Sweden; 12 UK Dementia Research Institute at UCL, London, UK; 13 Hong Kong Center for Neurodegenerative Diseases, Hong Kong, China; 14 Wisconsin Alzheimer’s Disease Research Center, University of Wisconsin School of Medicine and Public Health, University of Wisconsin, University of Wisconsin-Madison, Madison, WI USA; 15 InFLAMES Research Flagship Center, University of Turku, Turku, Finland

**Keywords:** Alzheimer’s disease, Blood biomarkers, PET imaging

## Abstract

**Background:**

Alzheimer’s disease (AD) is characterized by the accumulation of amyloid-β (Aβ) plaques, neurofibrillary tau tangles, and neurodegeneration in the brain parenchyma. Here, we aimed to (i) assess differences in blood and imaging biomarkers used to evaluate neurodegeneration among cognitively unimpaired *APOE* ε4 homozygotes, heterozygotes, and non-carriers with varying risk for sporadic AD, and (ii) to determine how different cerebral pathologies (i.e., Aβ deposition, medial temporal atrophy, and cerebrovascular pathology) contribute to blood biomarker concentrations in this sample.

**Methods:**

Sixty *APOE* ε4 homozygotes (*n* = 19), heterozygotes (*n* = 21), and non-carriers (*n* = 20) ranging from 60 to 75 years, were recruited in collaboration with Auria biobank (Turku, Finland). Participants underwent Aβ-PET ([^11^C]PiB), structural brain MRI including T1-weighted and T2-FLAIR sequences, and blood sampling for measuring serum neurofilament light chain (NfL), plasma total tau (t-tau), plasma N-terminal tau fragments (NTA-tau) and plasma glial fibrillary acidic protein (GFAP). [^11^C]PiB standardized uptake value ratio was calculated for regions typical for Aβ accumulation in AD. MRI images were analysed for regional volumes, atrophy scores, and volumes of white matter hyperintensities. Differences in biomarker levels and associations between blood and imaging biomarkers were tested using uni- and multivariable linear models (unadjusted and adjusted for age and sex).

**Results:**

Serum NfL concentration was increased in *APOE* ε4 homozygotes compared with non-carriers (mean 21.4 pg/ml (SD 9.5) vs. 15.5 pg/ml (3.8), *p* = 0.013), whereas other blood biomarkers did not differ between the groups (*p* > 0.077 for all). From imaging biomarkers, hippocampal volume was significantly decreased in *APOE* ε4 homozygotes compared with non-carriers (6.71 ml (0.86) vs. 7.2 ml (0.7), *p* = 0.029). In the whole sample, blood biomarker levels were differently predicted by the three measured cerebral pathologies; serum NfL concentration was associated with cerebrovascular pathology and medial temporal atrophy, while plasma NTA-tau associated with medial temporal atrophy. Plasma GFAP showed significant association with both medial temporal atrophy and Aβ pathology. Plasma t-tau concentration did not associate with any of the measured pathologies.

**Conclusions:**

Only increased serum NfL concentrations and decreased hippocampal volume was observed in cognitively unimpaired APOEε4 homozygotes compared to non-carriers. In the whole population the concentrations of blood biomarkers were affected in distinct ways by different pathologies.

## Introduction

Alzheimer’s disease (AD) is the most common cause of dementia, and it is estimated that over 400 million individuals worldwide are in the AD continuum which constituents a decade long asymptomatic phase [1]. AD is characterized by the accumulation of amyloid-β (Aβ) plaques and neurofibrillary tau tangles in the brain parenchyma, accompanied by neurodegeneration [2]. Hereditary factors play a significant role in the prevalence of AD [3], and the epsilon 4 allele of apolipoprotein E gene (*APOE* ε4) is the strongest genetic risk factor associated with sporadic AD [4]. The *APOE* ε4 allele has a dose-dependent effect on the prevalence of AD, with individuals carrying one *APOE* ε4 allele having a three times greater risk for AD, and those with two *APOE* ε4 alleles having a risk 14.9 times higher when compared with individuals without *APOE* ε4 alleles [5]. *APOE* ε4 is well-known for increasing Aβ accumulation in AD [6, 7] and is linked to neurodegeneration in AD as well as other neurodegenerative diseases [8, 9]. Also, it has been found that the APOE ε4 allele affects gray matter volume in the brain, particularly reducing volume in the hippocampus and other regions associated with memory and executive functions [10]. These structural changes, observed even in healthy individuals, have suggested a dose-dependent vulnerability to neurodegeneration associated with the APOE ε4 allele. In addition to the risk of AD, *APOE* ε4 increases the risk of cardiovascular and cerebrovascular disease [11–13].

Current clinically validated biomarkers for AD require cerebrospinal fluid (CSF) sampling or brain imaging. In vivo detection of Aβ and tau pathologies involves CSF measurements (CSF Aβ42/40 and p-tau181, respectively) or the use of positron emission tomography (PET) imaging, while neurodegeneration is typically assessed through magnetic resonance imaging (MRI) or CSF total tau (t-tau) measurements [14]. However, these diagnostic methods are not universally available in all healthcare settings, can be associated with significant patient discomfort, and are often expensive. To address these challenges, the development of easily accessible blood-based biomarkers for AD has shown great promise, and many new biomarkers have recently been introduced [15], although they are not yet established or implemented in clinical practice.

Various blood biomarkers are already available for studying neurodegenerative disorders: CSF neurofilament light chain (NfL) and CSF t-tau are clinically used biomarkers of neuroaxonal injury and neurodegeneration that can also be measured in blood [16, 17]. Plasma NfL is increased in AD and in other neurodegenerative conditions [16], making it unspecific for any particular disease or condition [16, 17]. Effect sizes compared with neurologically healthy controls are lower in AD than in frontotemporal dementia (FTD), some parkinsonian disorders and amyotrophic lateral sclerosis (ALS) [16]. On the other hand, there are confounding effects of age [18, 19] and BMI [20] on plasma NfL levels. Furthermore, NfL levels are also linked to cerebrovascular pathologies [21] including white matter changes [22]. Plasma t-tau has been shown to be associated with neurodegeneration [23, 24], tau-PET [25], and cognitive functions [23, 24, 26], but its use is restricted by wide overlap between diagnostic conditions [27, 28]. To overcome this, other tau biomarkers targeting different epitopes have recently been introduced and studied in relation to neurodegeneration and AD [29–31]. One of the latest is a biomarker targeting soluble N-terminal tau fragments (NTA-tau). CSF NTA-tau concentrations are increased in AD and rapidly progressive neurodegenerative diseases, such as Creutzfeldt-Jakob disease (CJD) [23]. Additionally, higher plasma NTA-tau levels have been reported in symptomatic AD patients compared with controls, showing a stronger association with tau PET than Aβ PET or neurodegeneration [29, 32]. Finally, astrocytes are known to be activated in neurodegeneration [33] and in the presence of Aβ [34], and at present, it is possible to measure glial fibrillary acidic protein (GFAP), a marker of astrocytic activation, in blood. Previously, higher plasma GFAP concentration has been associated with the incidence and faster progression of AD [35–38].

Even though blood biomarkers have been extensively studied during recent years, comparisons evaluating subtle differences in both blood and imaging biomarker measurements between genetic risk groups are not available. In addition, since *APOE* ε4 is known to increase the risk of Aβ, tau, and vascular pathologies, it is important to further investigate which pathologies contribute to blood biomarker concentrations in these populations. In this study, we aimed to confirm previously reported differences in imaging biomarkers between the different *APOE* ε4 populations and to evaluate the associations of the aforementioned imaging and blood biomarkers in a cohort comprised of cognitively unimpaired individuals with varying *APOE* ε4-related genetic risk for sporadic AD. The objectives of our study were twofold: (1) To evaluate the differences in blood and imaging biomarkers among cognitively normal *APOE* ε4 homozygotes (ε4ε4), heterozygotes (ε4ε3), and non-carriers, and (2) to examine head-to-head within the entire cohort the extent to which different pathologies (Aβ, medial temporal atrophy, cerebrovascular pathology) explain the levels of different blood-based biomarkers.

## Materials and methods

### Study population

This study was part of a larger research project, and a detailed study protocol has been previously reported [30]. To summarize, the participants of this cross-sectional observational study were recruited in collaboration with the local Auria biobank. Main inclusion criteria were 60–75 years of age and a CERAD total score > 62 points at screening. Exclusion criteria were dementia or cognitive impairment, any neurological or psychiatric disease, diabetes, chronic inflammatory condition, and contraindication for MRI or PET imaging. Our cohort included subjects with one, two, or no *APOE* ε4 alleles, and all underwent brain PET and MRI imaging, and blood sample collection. The study was approved by the Ethical Committee of the Hospital District of Southwest Finland. All participants signed written informed consent.

### Blood biomarkers

Venous EDTA-plasma and serum samples were acquired after a 10–12 h fasting period as reported previously [30] according to in-house standard operating procedures. Samples were gently inverted 5–10 times, centrifuged (2200 × g, 10 min), aliquoted, and stored at − 80◦C prior to biomarker analysis.

All blood biomarkers were measured at the Clinical Neurochemistry Laboratory, University of Gothenburg (Mölndal, Sweden) using the Single molecule array method and HD-X analysers (Quanterix). Commercial kits provided by Quanterix were used to measure single analyte serum NfL (Simoa® NF-light™, #103,186, Quanterix), plasma GFAP (Simoa® GFAP discovery, #102,336, Quanterix), and plasma t-tau (Simoa® Tau Advantage, #101,552, Quanterix). Measurements were performed following the instructions provided by the manufacturer. Briefly, randomized samples were first thawed at room temperature, vortexed (2000 rpm, 10 min, at room temperature), centrifuged (4000 × g, 10 min, at room temperature) and plated. Internal quality control samples were included to the beginning and end of the plate. Calibrators and controls were analysed in duplicates, samples in singlicates. Plasma GFAP concentrations have been reported before [39], and were added here to further investigate the association of GFAP with the biomarkers.

Plasma NTA-tau was measured using an in-house assay, described in detail elsewhere [32, 40]. Briefly, the NTA assay targets soluble N-terminal tau fragments (phosphorylated and non-phosphorylated), and utilizes mouse monoclonal antibodies targeting amino acids (aa) 159–163 (HT7, #MN1000, ThermoScientific, used as a capture antibody) and aa 6–18 (Tau12, BioLegend, used as detector ). Sample handling was done as previously described, and after plating, samples were diluted 1:2 using the assay diluent (Tau 2.0, Quanterix). Calibrators and controls were always analysed as duplicates, samples as singlicates, and non-phosphorylated full-length Tau-441 (SignalChem) was used as an assay calibrator.

### Brain imaging

All subjects underwent a structural brain MRI including T1-weighted and T2-FLAIR sequences. Structural brain images were acquired by two different scanners, either with Philips Ingenuity 3.0 T TF PET/MRI (*n* = 38; Philips Healthcare, Amsterdam, the Netherlands) or Philips Ingenia 3.0 T (*n* = 22; Philips Healthcare, Amsterdam, the Netherlands).

MRI was used to acquire volumetric variables (hippocampal volume, parahippocampal volume and entorhinal volume), global cortical atrophy score, medial temporal lobe atrophy score, and total volume of white matter hyperintensities. To determine brain Aβ load, [^11^C]PiB PET scans (*n* = 60) were acquired from 40 to 90 min post injection (mean injected dose 497 [30] MBq) with an ECAT high-resolution research tomograph (HRRT, Siemens Medical Solutions, Knoxville, TN).

### Brain image analysis

PET and MRI image preprocessing and analysis were performed using an automated pipeline at Turku PET Centre [41], which executed the PET data frame by frame realignment, PET-MRI co-registration, FreeSurfer (Freesurfer v6, https://surfer.nmr.mgh.harvard.edu/) region of interest (ROI) parcellation and PET data kinetic modelling. Regional and voxel level [^11^C]PiB binding was quantified as standardized uptake value ratios (SUVR) calculated for 60 to 90 min post injection using the cerebellar cortex as the reference region. The Aβ PET results have been published earlier in more detail [39]. For this study, a composite neocortical [^11^C]PiB score was calculated as the volume weighted average of the [^11^C]PiB region-to-cerebellar cortex SUVRs for the lateral frontal, lateral temporal, and parietal cortices as well as the posterior cingulate, anterior cingulate, and precuneus. This composite [^11^C]PiB score was used to estimate brain Aβ load and its association with blood biomarkers.

The brain MRI images were also analysed using an automatic cNeuro image analysis tool (Combinostics Oy, Tampere, Finland) to extract detailed data for atrophy scores for different brain regions and volumes of white matter intensities [42–44]. Segmentation was done from T1- and T2-weighted MRI, and each voxel was labelled based on which region it belongs to, using multi-atlas segmentation of 133 regions [43]. The structural MRI images were analysed for i) volumes of different brain regions (hippocampus, parahippocampus and entorhinal cortex) and atrophy scores (global cortical atrophy and medial temporal lobe atrophy) as a proxy for neurodegeneration, and iii) volumes of white matter hyperintensities for estimation of existing cerebrovascular pathology. For volumetric analysis, we focused on the hippocampus, parahippocampus, and the entorhinal cortex due to their known association with neurodegeneration related to AD [45]. Global cortical atrophy score (continuous measure, range 0–3) was estimated computationally using voxel-based morphometry for the whole cortex and brain lobes [44]. Medial temporal lobe atrophy score (continuous measure, range 0–4) was estimated computationally from the volumes of the hippocampus and inferior lateral ventricle from the T1-weighted MR images, and an average was calculated from the left and right sides [44]. Volumes of white matter hyperintensities (ml) were segmented from T2-FLAIR images [42]. Voxel -based morphometry analysis was conducted from T1-weighted MRI using SPM12 (http://www.fil.ion.ucl.ac.uk/spm) running on MATLAB R2021b (Math-Works, Natick, MA, USA) to assess group differences in cerebral grey matter at the voxel level.

### Statistical analysis

Statistical analyses were performed using JMP Pro 17.0.0 (SAS Institute Inc., Cary, North Carolina, USA). All data following a normal distribution are presented as mean (standard deviation, SD), otherwise as median (interquartile range, IQR). The normality of the data was evaluated visually from the distribution and with the Shapiro-Wilk test. Differences in continuous variables (blood and imaging biomarker levels) between the three *APOE* groups were tested using linear regression models, adjusting for age and sex. If a significant effect was found, all pairs were compared using the *post hoc* Tukey-Kramer honest significance test for multiple comparisons. Correlations between the MRI variables and blood biomarker concentrations were evaluated using Spearman’s rank correlation in the whole sample and within the *APOE* groups.

In addition to group -level differences in biomarker concentrations, we further tested the effect of different pathologies (Aβ, medial temporal atrophy, cerebrovascular pathology) on the blood biomarker concentrations in our cognitively unimpaired sample enriched with *APOE* ε4 carriers. In all linear regression models, Aβ pathology was estimated by composite neocortical [^11^C]PiB SUVR, neurodegeneration by the medial temporal lobe atrophy score, and cerebrovascular pathology by total volume of white matter hyperintensities. During this project, two different MRI scanners were used for collecting data; thus, in all models that included MRI -derived variables, the MRI scanner was included as a covariate. First, we performed univariate independent regression models with each of the blood biomarkers (serum NfL, plasma NTA-tau, plasma t-tau, plasma GFAP) as a response variable and each pathology as a predictor (**Model 1**: Blood biomarker ∼ Pathology marker). Next, we ran the same models adjusting for age, sex and BMI (**Model 2**: Blood biomarker ∼ Pathology marker + age + sex + BMI). To determine if the different pathologies had an effect simultaneously, we combined all three pathology markers as predictors in the same model (**Model 3**: Blood biomarker ∼ All pathology markers). In the last model, we again added sex, age, and BMI as covariates to the multivariate model including all pathologies as predictors (**Model 4**: Blood biomarker ∼ All pathology markers + age + sex + BMI). The normality assumption was evaluated based on the residuals and confirmed visually and calculated using the Shapiro-Wilk test. If the normality assumption was not fulfilled the blood biomarker values were log transformed, after which normality assumption was met. Variance inflation factor values were used to check that the independent variables were not highly correlated with each other. Adjusted R-squared (R^2^) was used to evaluate how well the models fit the data.

Voxel-wise volumetric differences between the *APOE* groups were tested using linear regression in statistical parametric mapping (SPM12) to evaluate if structural differences were present also outside the *a priori* chosen brain regions. Uncorrected *p* < 0.001 combined with a cluster-level false discovery rate (FDR) correction for multiple comparisons was considered statistically significant in the voxel-based analyses. When significant FDR corrected clusters were found we applied family wise error (FWE) correction with *p* < 0.05 to see if the results survived the tighter threshold. Voxel wise regression analysis was also done with SPM12 to analyze correlations between the biomarkers and grey matter volumes in the whole study population. All voxel-wise analyses were adjusted for age, sex, MRI scanner, and total intracranial volume.

## Results

### Participant demographics

Demographic and descriptive data are presented in Table 1. One subject from the *APOE* ε4ε4 group was excluded from the analysis due to abnormal brain structure. No significant differences in age (*p* = 0.75), sex (*p* = 0.91), education (*p* = 0.37) or BMI (*p* = 0.86) were observed between the *APOE* groups. In the whole study population, age correlated positively with serum NfL concentration (Rho = 0.36, *p* = 0.0051) and global cortical atrophy score (Rho = 0.37, *p* = 0.0034). On the contrary, age correlated negatively with hippocampal volume (Rho = -0.30, *p* = 0.018) and total volume of white matter hyperintensities (Rho = -0.27 *p* = 0.033). Higher BMI correlated with lower serum NfL concentrations (Rho = -0.29, *p* = 0.024).

**Table 1:** Demographics and descriptive data of the APOE ε4 homozygotes, heterozygotes, and non-carriers included in the study

	APOE ε4ε4	APOE ε4ε3	APOE ε3ε3	*p*
n	18	21	20	
Age (y), mean (SD)	67.3 (4.74)	67.3 (4.90)	68.3 (4.55)	0.75
Sex (M/F), n(%)	7/12 (37/63)	7/14 (33/67)	8/12 (40/60)	0.91
Education, n (%)				0.37
Primary school	7 (37)	4 (19)	7 (35)	
Middle or comprehensive school	4 (21)	4 (19)	3 (15)	
High school	7 (37)	6 (29)	7 (35)	
College or university	1 (5)	7 (33)	3 (15)	
MMSE, median (IQR)	28 (27–29)	29 (28–30) *	29 (27–30)	**0.039**
BMI (kg/m2), mean (SD)	26.6 (4.48)	26.7 (3.46)	27.3 (4.96)	0.86
Serum NfL pg/ml, mean (SD)	21.1 (9.34)*	17.8 (7.19)	15.5 (3.83)	**0.011**
Plasma NTA, median (IQR)	0.1 (0.03–0.24)	0.1 (0.049–0.18)	0.14 (0.05–0.25)	0.65
Plasma t-tau, mean (SD)	1.48 (0.75)	1.52 (0.53)	1.46 (0.55)	0.52
Plasma GFAP, median (IQR)	186 (124–269)	150 (104–170)	128 (105–147)	0.077
[^11^C]PIB composite SUVR, median (IQR)	2.13(1.61–2.83)	1.55 (1.43–2.02)*	1.47 (1.38–1.66)*	**0.0024**
WMH (ml), median (IQR)	4.37 (2.58–10.45)	4.70 (3.06–5.97)	3.92 (1.89–8.71)	0.36
Medial temporal atrophy score, median (IQR)	0.08 (0–0.76)	0.09 (0–0.35)	0.01 (0–0.36)	0.53
Global cortical atrophy score, median (IQR)	0.12 (1.67^e − 7^–1.12)	0.05 (1.67^e − 7^–0.28)	0.04 (1.67^e − 7^–0.34)	0.52
Hippocampus volume (ml), mean (SD)	6.62 (0.92)	7.02 (0.79)	7.27 (0.70)*	**0.041**
Entorhinal volume (ml), mean (SD)	4.23 (0.65)	4.47 (0.38)	4.52 (0.36)	0.13
Parahippocampal volume (ml), mean (SD)	5.50 (0.71)	5.8 (0.52)	5.89 (0.60)	0.12

Data are presented as mean (standard deviation) or median (interquartile range) depending on the distribution. Differences between groups were tested with one-way ANOVA with Tukey’s honest significance test, or Kruskal-Wallis test with Steel-Dwass method for multiple comparisons for continuous variables. χ2 test was used for testing categorical variables. P-value presents overall difference between groups. Significant differences in pairwise comparisons to APOE ε4ε4 homozygotes (*) are also presented. Abbreviations: BMI, body mass index; MMSE, mini-mental state examination; SUVR, standardized uptake value ratio; Serum NfL, Neurofilament light; Plasma NTA, N-terminal tau; Plasma t-tau, Quanterix total tau; Plasma GFAP, Glial fibrillary acidic protein; WMH, volumes of white matter hyperintensities

### Blood biomarkers across *APOE* groups

**Fig. 1:**
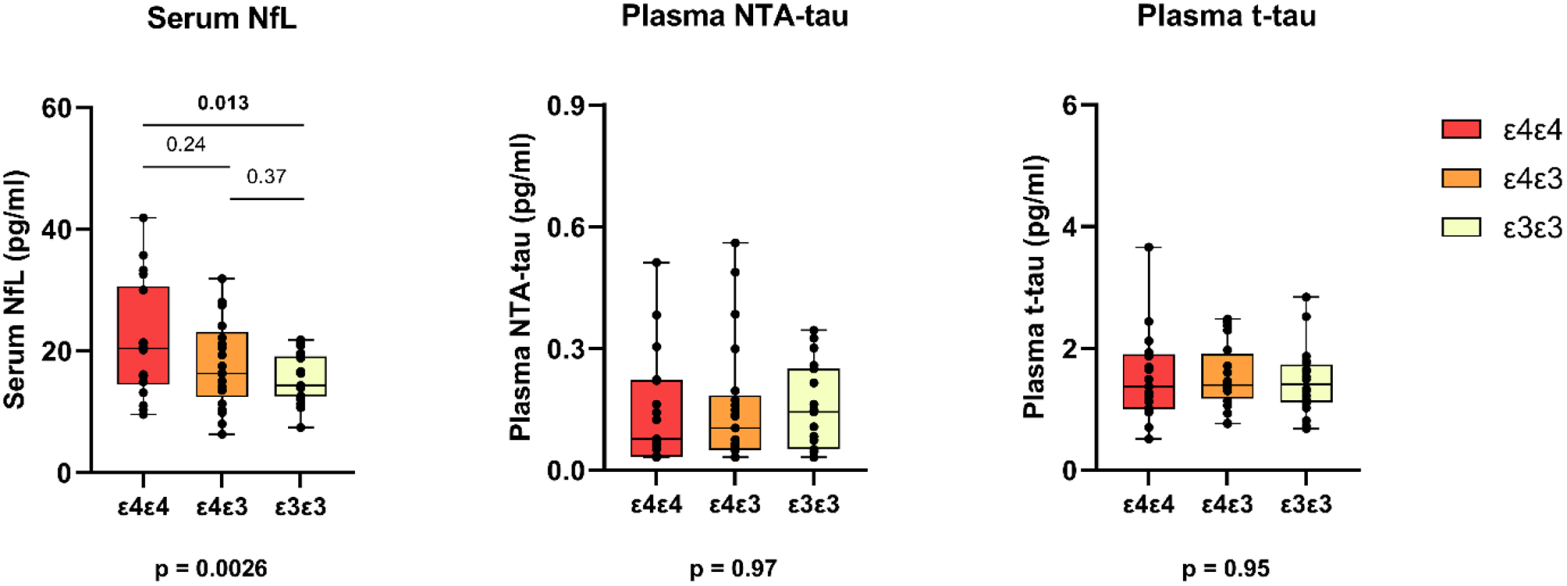
Serum NfL, plasma NTA-tau and plasma t-tau concentrations in cognitively unimpaired APOE ε4 homozygotes (ε4ε4), heterozygotes (ε4ε3) and noncarriers (ε3ε3). Raw concentrations, median, first and third quartile and range are presented by the box plot, p values are further adjusted for age and sex. P-values below the figures presents overall difference between groups

Statistically significant differences between the *APOE* groups were found for serum NfL (*p* = 0.018), but not for plasma NTA-tau (*p* = 0.97), plasma t-tau (*p* = 0.95) or plasma GFAP (*p* = 0.077) (Fig. 1). Post hoc comparisons between groups showed that serum NfL concentrations were significantly higher in the *APOE* ε4ε4 (21.4 pg/ml (9.5); mean (SD)) compared with non-carriers (15.5 pg/ml (3.8)) (*p* = 0.013, Tukey HSD), whereas differences between the *APOE* ε4ε3 (17.8 pg/ml (7.2)) and *APOE* ε4ε4 (*p* = 0.24) or non-carriers (*p* = 0.37) were not statistically significant.

### Structural MRI findings across *APOE* groups

**Fig. 2:**
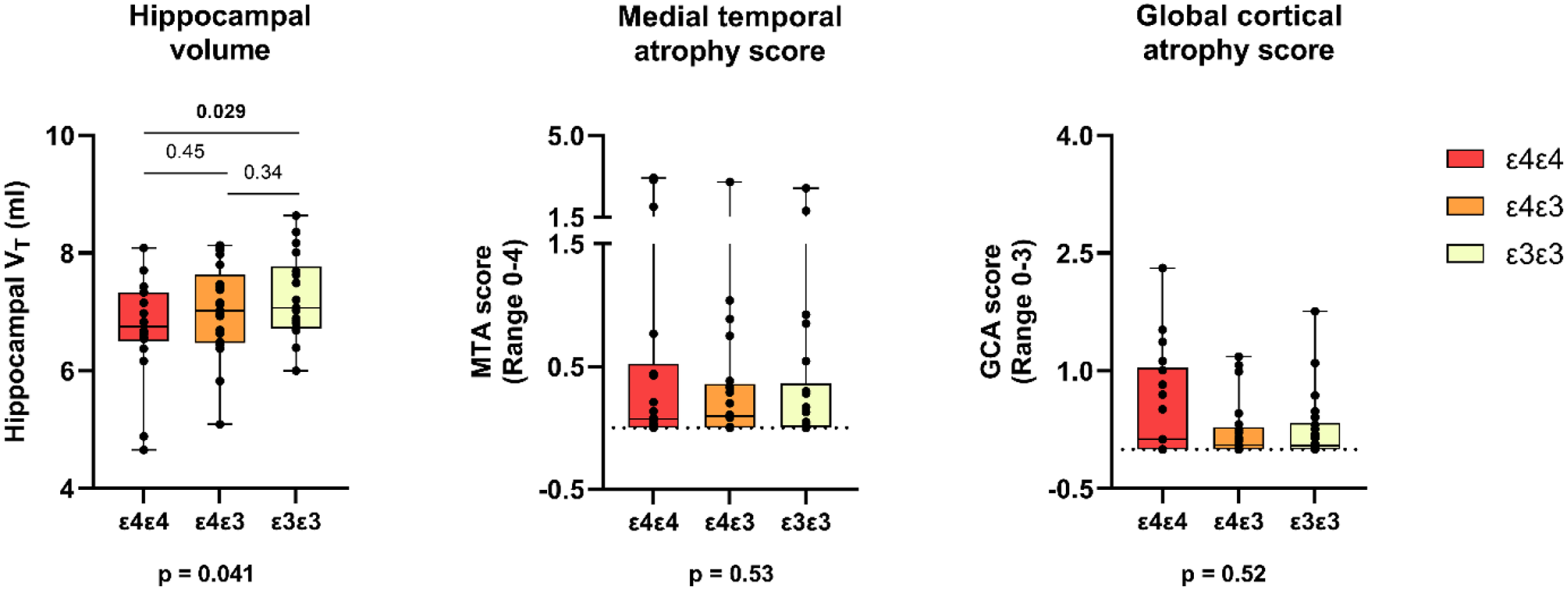
Hippocampal volume, medial temporal and global atrophy scores in cognitively unimpaired APOE ε4 homozygotes (ε4ε4), heterozygotes (ε4ε3) and noncarriers (ε3ε3). Raw concentrations, median, first and third quartile and range are presented by the box plot, p values are further adjusted for age and sex. P-values below the figures presents overall difference between groups

For imaging markers of neurodegeneration, statistically significant differences among the *APOE* groups were found only for hippocampal volume (*p* = 0.041), that was significantly lower in the *APOE* ε4ε4 (6.71 ml, 0.86; mean, SD) compared with non-carriers (7.2 ml, 0.7, *p* = 0.029) (Fig. 2). No differences were present between the *APOE* ε4ε4 and *APOE* ε4ε3 (7.0 ml, 0.80, *p* = 0.45) or between the *APOE* ε4ε3 and non-carriers (*p* = 0.34). No significant *APOE* group differences were seen in global cortical atrophy score (*p* = 0.52), medial temporal lobe atrophy score (*p* = 0.52), volumes of white matter hyperintensities (*p* = 0.36), entorhinal volume (*p* = 0.13) or parahippocampal volume (*p* = 0.12).

**Fig. 3:**
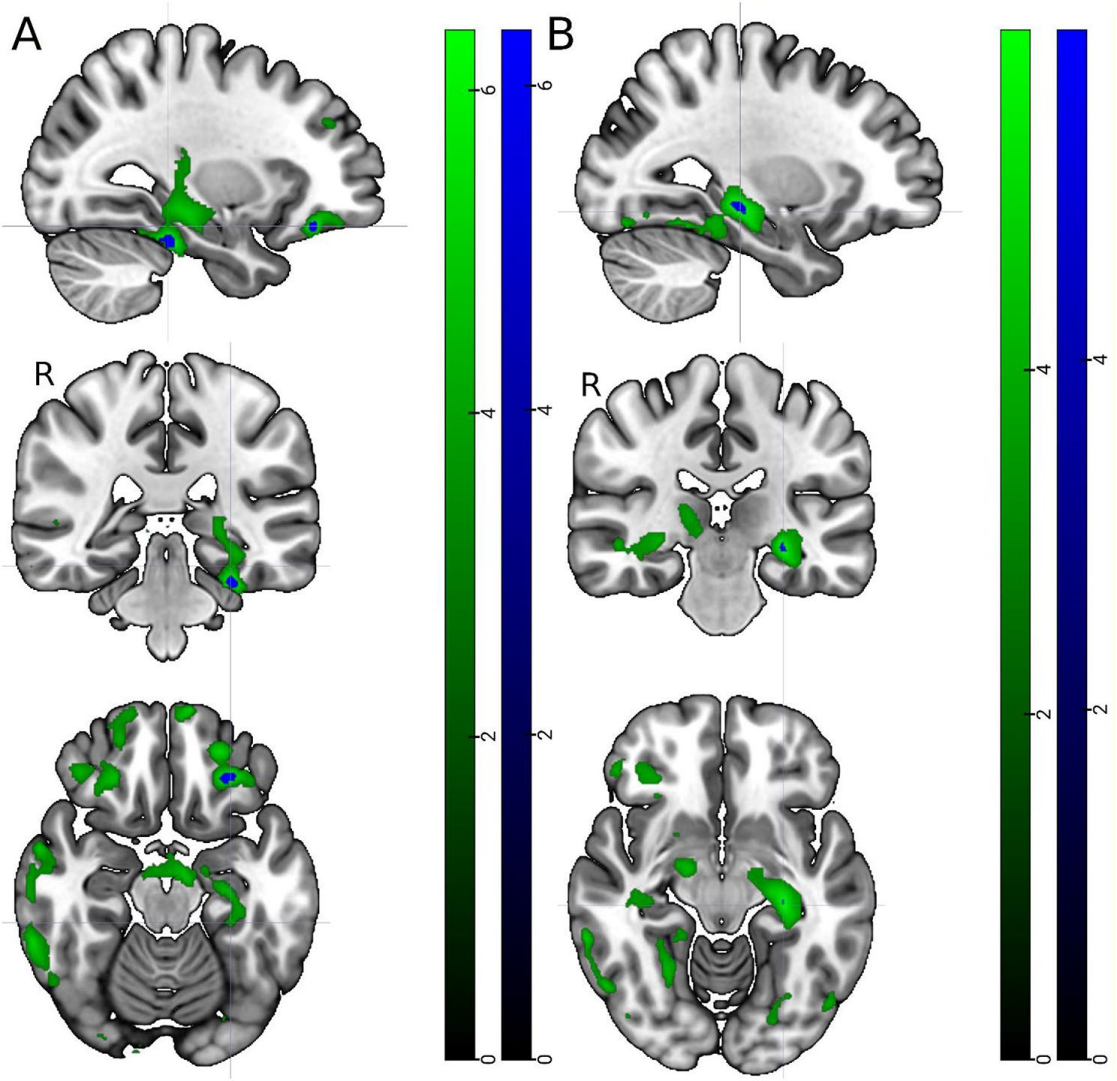
Significant MRI voxel based morphometry results. (**A**) ε4ε4 < ε4ε3 (**B**) ε4ε4 < ε3ε3

We then further analysed differences in grey matter volume between the *APOE* groups using voxel-based morphometry. When the significance threshold was set to FWE corrected *p* < 0.05, lower volume was found in the left hippocampal region of *APOE* carriers compared with non-carriers (Fig. 3, blue scale). Also, between the ε4ε4 and ε4ε3 carriers, there were similar findings, but also small clusters in the frontal cortex. With a more lenient threshold of FDR corrected *p* < 0.001, broader reductions in grey matter volumes were found mainly in the hippocampal and parahippocampal regions of *APOE* ε4ε4 carriers compared with non-carriers (Fig. 3, green scale). Similar findings were also between the ε4ε4 and ε4ε3 carriers, but also in the frontal and right lateral areas.

### Correlations between imaging and blood biomarkers

**Fig. 4:**
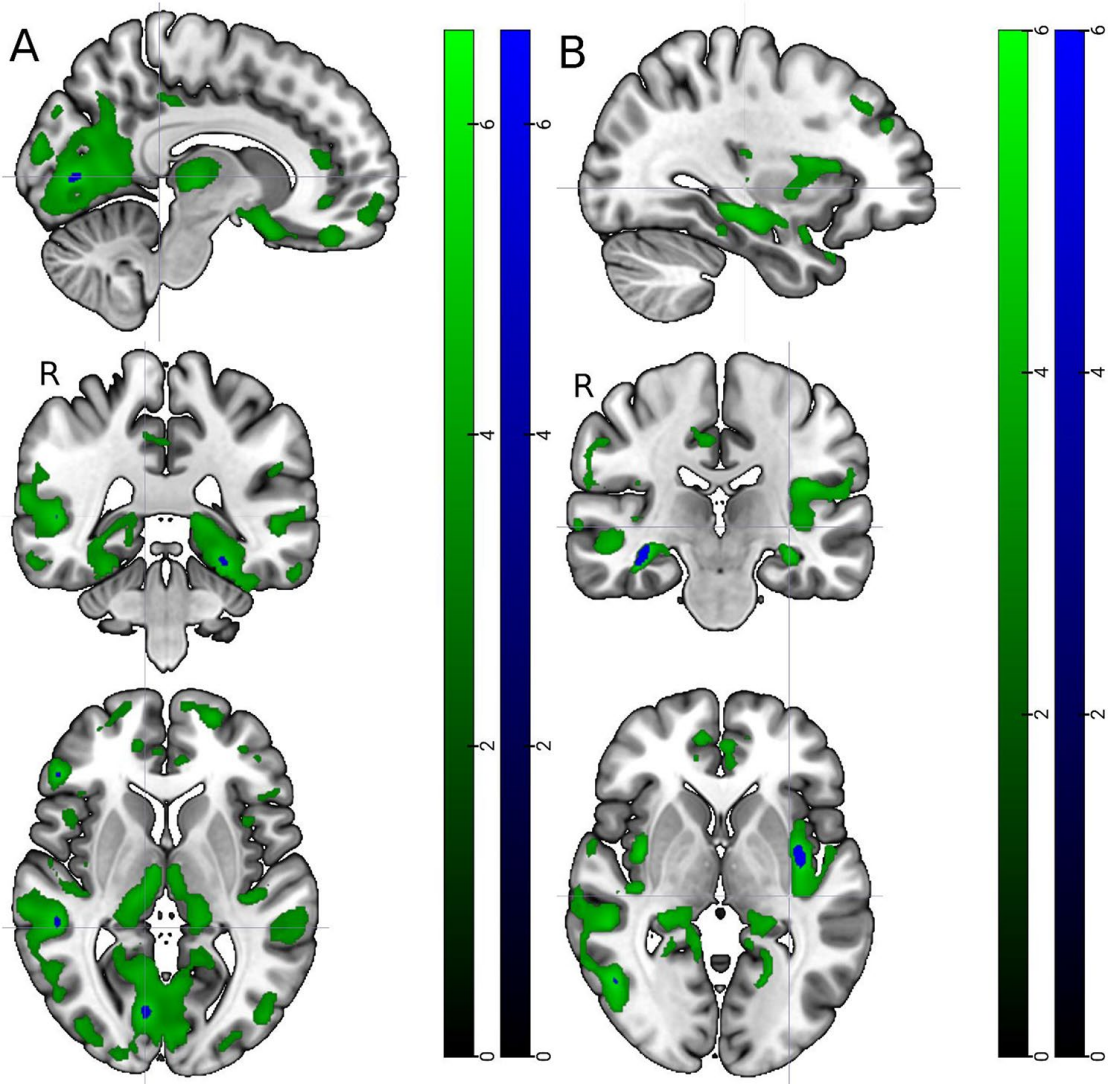
Voxel wise correlations between (**A**) serum NfL, (**B**) plasma GFAP and smaller grey matter volume. FDR corrected *p* < 0.001 in green colour. FWE corrected *p* < 0.05 in blue colour

In the whole study population, higher global cortical atrophy score correlated with higher serum NfL concentrations (Rho = 0.40, *p* = 0.0017). We then performed additional exploratory analysis stratifying by the *APOE* group and found that all correlations were significant only in the *APOE* ε4ε4 group. In the *APOE* ε4ε4 group, higher global cortical atrophy score correlated with higher serum NfL (Rho = 0.78, *p* < 0.001) and higher plasma GFAP concentrations (Rho = 0.53, *p* = 0.024). Higher medial temporal atrophy score correlated with both higher plasma NTA-tau (Rho = 0.61, *p* = 0.0097) and serum NfL concentrations (Rho = 0.63, *p* = 0.0049). No significant correlations were seen in the *APOE* ε4ε3 group or non-carriers. In addition to the ROI based imaging results, we did an exploratory voxel based regression analysis in the whole study population to see if the biomarkers had associations outside the ad hoc chosen brain areas. Here, the significant correlations to smaller grey matter volume were with serum NfL and plasma GFAP (Fig. 4). Serum NfL had negative correlations with grey matter volume in all cortical regions (Fig. 4A). Plasma GFAP had negative correlations with grey matter volume in left insular cortex and right hippocampus (Fig. 4B).

### Effect of Aβ, medial temporal atrophy, cerebrovascular pathology on blood biomarker concentrations

Next, we tested in the whole cohort, what proportion of the variation in blood biomarker concentrations was explained by Aβ pathology (estimated with [^11^C]PiB PET), cerebrovascular pathology (estimated with total white matter lesion volume) and medial temporal. Blood biomarkers were set as response variables and pathology markers as predictors in all linear regression models. The blood biomarker values were log transformed, so that the models residuals fulfilled the normal assumption.

In univariate unadjusted (Model 1) and adjusted regression models (Model 2), we first looked at the effect of each pathology separately to a single blood biomarker (serum NfL, plasma NTA-tau, plasma t-tau, plasma GFAP). Cerebrovascular pathology had a positive association with serum NfL concentrations in Model 1 (R^2^ = 0.18, *p* = 0. 017, Table 2) and in Model 2, when further adjusting for age, sex, and BMI (R^2^ = 0.33, *p* = 0.021, Table 3). Medial temporal atrophy had a similar positive association with serum NfL concentrations in univariate Model 1 (R^2^ = 0.14, *p* = 0.025, Table 2) and in adjusted Model 2 (R^2^ = 0.32, *p* = 0.049, Table 3).

**Table 2:** Model 1 Linear regression results between a blood biomarker and an imaging biomarker

	Serum NfL			Plasma NTA			Plasma t-tau			Plasma GFAP		
Predictors	Estimate	R^2^	p	Estimate	R^2^	p	Estimate	R^2^	p	Estimate	R^2^	p
WMH, slope (95% CI)	**0.011 (0.0044 to 0.019)**	**0.18**	**0.017**	0.0047(-0.012 to 0.022)	0.072	0.62	-0.004 (-0.012 to 0.0036)	0.075	0.37	0.0064 (-0.0023 to 0.015)	0.0066	0.20
MTA, slope (95% CI)	**0.16 (0.038 to 0.28)**	**0.14**	**0.025**	**0.42 (0.13 to 0.71)**	**0.19**	**0.026**	0.063 (-0.045 to 0.21)	0.082	0.29	**0.20 (0.067 to 0.34)**	**0.11**	**0.022**
PiB, slope (95% CI)	0.15 (-0.029 to 0.32)	0.030	0.15	0.16 (-0.26 to 0.58)	-0.0080	0.53	0.074 (-0.12 to 0.27)	-0.0075	0.52	**0.26 (0.069 to 0.46)**	**0.10**	**0.022**

Serum NfL = neurofilament light, Plasma NTA = N-terminal tau marker, Plasma t-tau = Quanterix total tau marker, Plasma GFAP = glial fibrillary acidic protein, WMH = volumes of white matter hyperintensities, MTA = medial temporal lobe atrophy score, PiB = [^11^C]PiB composite score. Blood biomarker values are log transformed. WMH and MTA adjusted for MRI scanner. P < 0.05 are bolded. P-values are FDR-corrected including all p-values from tables 1, 2, 3, and 4. Blood biomarker values were log transformed

Medial temporal atrophy was also positively associated with plasma NTA-tau levels both in the unadjusted (R^2^ = 0.19, *p* = 0.026, Table 2) and adjusted model (R^2^ = 0.15, *p* = 0.022, Table 3).

**Table 3:** Model 2 adjusted linear regression results between a blood biomarker and an imaging biomarker

	Serum NfL			Plasma NTA			Plasma t-tau			Plasma GFAP		
Predictors	Estimate	R^2^	p	Estimate	R^2^	p	Estimate	R^2^	p	Estimate	R^2^	p
WMH, slope (95% CI)	**0.0088 (0.0019 to 0.016)**	**0.33**	**0.021**	0.007 (-0.011 to 0.025)	0.027	0.54	-0.004 (-0.012 to 0.0037)	0.11	0.37	0.0037 (-0.0054 to 0.013)	0.015	0.51
MTA, slope (95% CI)	**0.13 (0.019 to 0.24)**	**0.32**	**0.049**	**0.44 (0.13 to 0.76)**	**0.15**	**0.022**	-0.12 (-0.010 to 0.25)	0.14	0.13	**0.22 (0.075 to 0.36)**	**0.15**	**0.022**
PiB, slope (95% CI)	0.14 (-0.026 to 0.30)	0.20	0.16	0.12 (-0.33 to 0.56)	-0.054	0.62	0.05 (-0.15 to 0.25)	0.045	0.61	**0.27 (0.073 to 0.47)**	**0.13**	**0.022**

Serum NfL = neurofilament light, Plasma NTA = N-terminal tau marker, Plasma t-tau = Quanterix total tau marker, Plasma GFAP = glial fibrillary acidic protein, WMH = volumes of white matter hyperintensities, MTA = medial temporal lobe atrophy score, PiB = [^11^C]PiB composite score. Blood biomarker values are log transformed. Results adjusted for Age, Sex and BMI. WMH and MTA also adjusted for MRI scanner. Significant values are bolded. P-values are FDR-corrected including all p-values from tables 1, 2, 3, and 4. Blood biomarker values were log transformed

In addition, medial temporal atrophy had a significant effect on plasma GFAP levels in both the unadjusted (R^2^ = 0.11, *p* = 0.022, Table 2) and adjusted model (R^2^ = 0.15, *p* = 0.022, Table 3). Aβ pathology was positively associated only with plasma GFAP levels, both alone (R^2^ = 0.10, *p* = 0.022, **Table 2**) and when adjusted for demographic variables (R^2^ = 0.13, *p* = 0.022, Table 3).

None of the pathology markers significantly explained variance in plasma t-tau concentrations (Tables 2 and 3).

In the multivariate linear regression models (Model 3 and Model 4, **Tables 4 and 5**), when we combined all three pathology markers as predictors, the results were aligned with the previously presented models. For serum NfL, both models were statistically significant (R^2^ = 0.22, *p* = 0.017 for Model 3, and R^2^ = 0.32, *p* < 0.001 for Model 4 including covariates) and in both models, cerebrovascular pathology was the predictor showing a significant effect on the outcome (Model 3, *p* = 0.0062; Model 4, *p* = 0.039). In Model 3, medial temporal atrophy also had a significant effect on serum NfL levels (*p* = 0.042), but this was dampened when further adjusting for age, sex, and BMI (*p* = 0.076).

For plasma NTA-tau, the Model 3 proved to be significant (R2 = 0.18, *p* = 0.021, Table 4), but when adjusting for age, sex and BMI the significance diminished (R2 = 0.14, *p* = 0.083, Table 5) In the Model 3 only medial temporal atrophy contributed significantly to these model (Model 3, *p* = 0.0063).

**Table 4:** Model 3 results for the blood biomarkers and the imaging biomarkers

	Serum NfL			Plasma NTA			Plasma t-tau			Plasma GFAP		
	**R**^**2**^ **= 0.22 p = 0.017**			**R**^**2**^ **= 0.18 p = 0.021**			R^2^ = 0.083 p = 0.14			**R**^**2**^ **= 0.18 p = 0.023**		
Predictors	Estimate	F ratio	Std β	Estimate	F ratio	Std β	Estimate	F ratio	Std β	Estimate	F ratio	Std β
WMH, slope (95% CI)	0.0098 (0.0029 to 0.017)**	**8.12**	**0.34**	0.0015 (-0.015 to 0.022)	0.032	0.022	-0.0047 (-0.012 to 0.0030)	1.51	-0.16	0.0039 (-0.0041 to 0.12)	0.95	0.12
MTA, slope (95% CI)	0.12 (0.0044 to 0.23)*	**4.34**	**0.25**	0.45 (0.13 to 0.76)**	**8.10**	**0.37**	0.09 (-0.042 to 0.22)	1.85	0.18	0.16 ( 0.020 to 0.29)*	**5.29**	**0.29**
PiB, slope (95% CI)	0.058 (-0.11 to 0.23)	0.85	0.11	-0.11 (-0.51 to 0.76)	0.28	-0.068	0.017 (-0.17 to 0.21)	0.032	0.025	0.22 (0.028 to 0.42)*	**5.27**	**0.29**

Serum NfL = neurofilament light, Plasma NTA = N-terminal tau marker, Plasma t-tau = Quanterix total tau marker, Plasma GFAP = glial fibrillary acidic protein, WMH = volumes of white matter hyperintensities, MTA = medial temporal lobe atrophy score, PiB = [^11^C]PiB composite score, Blood biomarker values are log transformed, Results adjusted for MRI scanner. P-values are FDR-corrected including all p-values from tables 1, 2, 3, and 4. Significant values are bolded

Blood biomarker values were log transformed. *p < 0.05. **p < 0.01. ***p < 0.001

**Table 5:** Model 4 adjusted results for the blood biomarkers and the imaging biomarkers

	Serum NfL			Plasma NTA			Plasma t-tau			Plasma GFAP		
	**R**^**2**^ **= 0.32 p = 0.0064**			**R**^**2**^ **= 0.14 p = 0.083**			**R**^**2**^ **= 0.14 p = 0.082**			**R**^**2**^ **= 0.21 p = 0.020**		
Predictors	Estimate	F ratio	Std β	Estimate	F ratio	Std β	Estimate	F ratio	Std β	Estimate	F ratio	Std β
WMH, slope (95% CI)	0.0073 (0.00038 to 0.014)*	**4.48**	**0.25**	0.0024 (-0.016 to 0.020)	0.073	0.037	-0.0057 (-0.014 to 0.0023)	2.02	-0.19	0.00043 (-0.0080 to 0.089)	0.011	0.013
MTA, slope (95% CI)	0.10 (-0.011 to 0.21)	3.27	0.21	0.45 (0.12 to 0.79)	7.60	0.38	0.13 (-0.0024 to 0.27)	3.90	0.27	0.18 (0.041 to 0.32)*	**6.70**	**0.33**
PiB, slope (95% CI)	0.070 (-0.087 to 0.23)	0.38	0.10	-0.092 (-0.51 to 0.33)	0.19	-0.059	0.014 (-0.18 to 0.21)	0.022	0.020	0.23 (0.043 to 0.43)*	**6.04**	**0.31**

Serum NfL = neurofilament light, Plasma NTA = N-terminal tau marker, Plasma t-tau = Quanterix total tau marker, Plasma GFAP = glial fibrillary acidic protein, WMH = volumes of white matter hyperintensities, MTA = medial temporal lobe atrophy score, PiB = [^11^C]PiB composite score, Blood biomarker values are log transformed, Results adjusted for Age, Sex, BMI and MRI scanner. P-values are FDR-corrected including all p-values from tables 1, 2, 3, and 4. Significant values are bolded. Blood biomarker values were log transformed. *p < 0.05. **p < 0.01. ***p < 0.001

For plasma GFAP, both models were significant (R^2^ = 0.18, *p* = 0.023 and R^2^ = 0.21, *p* = 0.020, Tables 4 and 5). In both models, Aβ pathology and medial temporal atrophy had a similar significant effect on plasma GFAP concentration (Model 3, *p* = 0.025 and *p* = 0.026; Model 4, *p* = 0.013 and *p* = 0.018 for medial temporal lobe atrophy score, [^11^C]PiB SUVR, respectively).

Last, in the models explaining plasma t-tau concentrations neither Model 3 or Model 4 were not significant (R^2^ = 0.081, *p* = 0.14 and R^2^ = 0.14, *p* = 0.082, Tables 4 and 5).

## Discussion

In this study, we examined early differences in blood biomarker concentrations, cortical volumes, white matter hyperintensities and grey matter atrophy scores, which serve as biomarkers of neurodegeneration, in a cohort of cognitively unimpaired participants with different numbers of *APOE* ε4 alleles, and thereby varying risk for sporadic AD. Additionally, we investigated how the different pathologies (cerebrovascular pathology, medial temporal atrophy and Aβ pathology) explained the concentrations of blood biomarkers, using the entire cognitively unimpaired sample enriched with *APOE* ε4 carriers. Our main findings revealed that serum NfL concentrations were significantly increased, and hippocampal volume was significantly decreased in cognitively unimpaired *APOE* ε4 homozygotes compared with non-carriers. Second, in the whole sample, we observed different associations of cerebrovascular pathology, medial temporal atrophy and Aβ deposition with the blood biomarkers used to estimate neurodegeneration. Specifically, serum NfL levels were associated with cerebrovascular pathology and medial temporal atrophy, whereas plasma NTA-tau was associated only with medial temporal atrophy. Lastly, plasma GFAP was associated with medial temporal atrophy and Aβ pathology, while plasma t-tau levels could not be explained by any of the assessed pathologies.

The hippocampus is one of the first brain regions where structural changes become visible in the AD *continuum* [46]. APOE ε4 allele has been shown to affect gray matter volume in the brain in a dose dependent manner, particularly reducing volume in the posterior hippocampus and other regions associated with memory and executive functions [10]. In our study we found similar volumetric differences between *APOE4* gene dose groups, in the hippocampus. While ROI analysis revealed a statistically significant difference only between the *APOE* ε4ε4 group and non-carriers in hippocampal volumes, a visible linear trend related to *APOE* ε4 allele burden existed across the groups. This finding was further supported by the VBM analysis, which showed a significant difference in hippocampal volume between the *APOE* ε4ε4 group and non-carriers even when using a strict FWE-corrected threshold, and exhibited a difference between the *APOE* ε4ε4 and ε4ε3 groups with FDR-corrected threshold. Our findings agree with previous results regarding the loss of hippocampal volume in healthy *APOE* ε4 carriers [10, 47]. Furthermore, in individuals with mild cognitive impairment (MCI) or AD, hippocampal volume decreases have been observed in relation to *APOE* ε4 status [48–50].

NfL concentrations measured from blood are elevated in AD and other neurodegenerative diseases [16, 17]. Here we found serum NfL concentrations to be elevated already in cognitively unimpaired *APOE* ε4ε4 carriers compared with non-carriers. This finding is consistent with a recent study that reported differences between cognitively unimpaired non-carriers and both the *APOE* ε4ε4 and ε4ε3 groups [51]. Previously significant differences in CSF NfL concentration between *APOE* ε4 carriers and non-carriers from prodromal AD patients has been reported [52]. In contrast to our findings, Mielke (2019) did not observe any effect of *APOE* ε4 on plasma or CSF NfL levels in cognitively unimpaired subjects [53]. However, the *APOE* ε4 status was reported only as positive or negative, categorizing subjects as carriers if they had at least one *APOE* ε4 allele, whereas we evaluated homozygotic and heterozygotic *APOE4* carriers separately. Additionally, most subjects were non-carriers, whereas in our study, the different *APOE* groups were well balanced. If we use the binarized, *APOE* ε4 positive and negative categorisation, the difference between *APOE* ε4 carriers and non-carriers in our sample is still statistically significant (*p* = 0.035).

Higher serum NfL correlated with cortical atrophy and voxel-wise regression analysis were in line with this finding. When we examined this association further with multiple regression models, we found that higher serum NfL concentration was associated with cerebrovascular pathology, estimated from the volume of white matter hyperintensities, and neurodegeneration in the medial temporal lobe. The effect of cerebrovascular pathology seemed to be stronger as it was the only explaining predictor in the model with all pathologies and demographic covariates. This concurs with a study in an elderly population with high prevalence of cerebral small vessel disease burden where plasma NfL was associated with both neurodegenerative and vascular pathologies [54]. White matter hyperintensities are considered markers of cerebral small vessel disease, indicating increased water content and mobility, demyelination, and axonal loss [55], while NfL is a nonspecific marker of axonal damage [16, 56]. Previous studies have demonstrated an increase in CSF NfL levels with an increased number of white matter hyperintensities [57–59]. Similar associations have been observed with plasma NfL in neuropathologically confirmed AD [60], AD dementia [61] and serum NfL in neurologically unaffected individuals [18]. These studies have also shown a significant influence of age on blood NfL levels, whereas in our study, the association of white matter hyperintensities with NfL was independent of age. NfL levels are known to rise with age [18, 19], but compared with the previous studies, our population was in a somewhat narrow age range (67 (4.7) years) and was enriched with *APOE* ε4ε4 carriers. *APOE* ε4 is associated with increased white matter hyperintensities [13, 62] so in this population, the effect of white matter hyperintensities on serum NfL concentration appeared to be more significant than age. Previous findings suggest that plasma NfL increases in response to amyloid-related neuronal injury in preclinical stages of Alzheimer’s disease, but is related to tau-mediated neurodegeneration in symptomatic patients [22]. However, in our analyses, Aβ-PET did not show any association with serum NfL concentrations, and the biomarker levels were primarily explained by the extent of cerebrovascular pathology. In addition to the association between white matter hyperintensities and NfL, we also found that, neurodegeneration, as estimated here by the medial temporal lobe atrophy score, was associated with higher serum NfL concentrations. In previous studies, plasma NfL has been associated with decreased volumes in the temporal cortex and hippocampal volume in subjects without dementia [63], and CSF NfL to decrease in whole brain and hippocampus volumes [64]. However, this association was weakened when adjusting for simultaneous effects of different pathologies and demographic variables.

NTA-tau is a novel biomarker of soluble, N-terminal tau fragments, recently suggested to be more related to tau tangle pathology (as estimated by tau PET), than Aβ-PET or neurodegeneration [32]. Here, we demonstrated an association between neurodegeneration and NTA-tau in a cognitively normal sample, enriched with “at-risk” individuals [40]. In CSF, NTA-tau has previously been found to be increased in CSF Aβ-positive individuals with MCI due to AD and AD. Similarly, plasma NTA-tau concentrations have been shown to be elevated in AD dementia compared with controls in a clinical pilot cohort [40]. In a study evaluating plasma NTA-tau levels along the AD continuum, it was observed that NTA-tau exhibited a stronger association with tau-PET compared with Aβ -PET. Additionally, plasma NTA-tau predicted tau-PET accumulation in middle to late Braak regions and neurodegeneration in the medial temporal lobe. These findings suggest that NTA-tau could serve as a tool for detecting and monitoring pathological changes associated with middle to late stages of AD [32]. Interestingly, in our study, we found that NTA-tau is associated with medial temporal lobe atrophy already in cognitively unimpaired subjects. This finding may be explained by the fact that our cohort is highly enriched with homozygous *APOE* ε4 carriers. *APOE* ε4 is known to be linked to elevated tau accumulation in the medial temporal lobe, and tau itself is an independent driver of neurodegeneration [65–70].

In our study, plasma total-tau (t-tau) did not exhibit any correlation with the neuroimaging biomarkers, unlike NTA-tau. Though plasma t-tau was among the earliest blood-based immunoassays used to detect soluble tau in AD, its diagnostic value is limited due to significant overlap in its levels among different diagnostic categories [26, 27]. While CSF t-tau does reflect the intensity of neuronal and axonal degeneration and damage in the brain [71], blood-based t-tau may also arise from peripheral sources, which diminishes its specificity for neurological conditions [72, 73]. Additionally, recent research suggests that only approximately 20% of plasma t-tau originates from the CNS, further challenging its effectiveness as a marker for brain pathology [74].

Plasma GFAP is a protein constituent of astrocyte intermediate filaments and elevated GFAP concentrations are thought to reflect astrocyte activation and reactive gliosis linked with several CNS disorders e.g. neurotrauma, ischemic stroke or neurodegenerative diseases [75]. In our study, we found that GFAP was the only blood biomarker associated with the level of Aβ pathology estimated by PET. This is in line with previous findings where higher plasma GFAP concentrations have been associated with higher Aβ load determined using PET imaging [37, 38, 76, 77] and in genetic forms of AD [78, 79]. Astrocytes are activated and express GFAP in the context of neurodegeneration [33] and in the presence of Aβ [34]. In AD, higher plasma GFAP levels have been associated with the incidence and faster progression of AD [35–38]. Plasma GFAP is also able to differentiate Aβ-positive and Aβ-negative cognitively unimpaired individuals [35, 37, 38, 76]. Here, also medial temporal atrophy was associated with higher plasma GFAP concentrations. Also, with the voxel-wise analysis, we saw association of higher plasma GFAP to reduced grey matter in the hippocampus. This is likely explained by activation of the astroglia by the subtle neurodegeneration present already in the medial temporal lobe of *APOE* ε4 carriers. Another explanation could be the type of astrocytes located in the medial temporal lobe as high GFAP expressing astrocytes have been found in the murine hippocampus [80, 81].

The strength of this study is in conducting a comparison of the impact of various pathologies on multiple blood biomarkers used to study neurodegenerative processes. Furthermore, it employs a meticulously selected population with a balanced distribution of subjects across the three *APOE*-genotypes. This approach enables a precise detection of the effects attributed to the *APOE* ε4 allele.

There are also limitations in this study. Firstly, it is important to note that the associations between the pathologies and blood biomarkers in the general population may differ from those observed in this study, as the sample used was heavily enriched with *APOE* ε4 carriers, a third of the sample comprising of individuals who were rare homozygotes. Additionally, the groups were relatively small for between group blood biomarkers comparisons, as the study’s initial power calculations were primarily focused on PET imaging biomarkers, and the small sample size might also limit the generalizability of our findings. Furthermore, the study lacked tau-PET and CSF tau measurements, which prevented the evaluation of the effect of tau-pathology on the blood biomarkers and would have greatly enriched the study.

## Conclusions

We investigated the differences associated with the *APOE* ε4 allele in both imaging and blood biomarkers of neurodegenerative disorders. Our findings revealed that individuals who were *APOE* ε4 homozygotes exhibited significant grey matter loss in the hippocampus, as well as elevated levels of serum NfL compared with non-carriers. Moreover, within our whole cohort, we demonstrated that Aβ deposition, cerebrovascular pathology, and medial temporal atrophy have distinct influences on the concentrations of the examined blood biomarkers. This study significantly contributes to our understanding of the relationship between the *APOE* ε4 gene, blood biomarkers, and imaging-based pathology markers in the early stages of the AD pathologic continuum. These findings have the potential to facilitate the development of novel diagnostic and prognostic tools for neurodegenerative diseases, particularly in the preclinical phase.

## Data Availability

The data are available from the corresponding author on reasonable request.

## Abbreviations

Aβ: Beta-amyloid
AD: Alzheimer’s disease
ALS: Amyotrophic lateral sclerosis
APOE: Apolipoprotein E gene
BMI: Body mass index
CNS: Central nervous system
CSF: Cerebrospinal fluid
FTD: Frontotemporal dementia
GFAP: Glial fibrillary acidic protein
NfL: Neurofilament light chain
NTA-tau: N-terminal tau fragments
MRI: Magnetic resonance imaging
MTA: Medial temporal lobe atrophy
PET: Positron emission tomography
ROI: Region of interest
Simoa: Single molecule array
SUVR: Standardized uptake value ratio
t-tau: total tau
VBM: Volume based morphometry
WMH: White matter hyperintensities
